# Similar antibody responses against SARS-CoV-2 in HIV uninfected and infected individuals on antiretroviral therapy during the first South African infection wave

**DOI:** 10.1093/cid/ciab758

**Published:** 2021-09-02

**Authors:** Jumari Snyman, Shi-Hsia Hwa, Robert Krause, Daniel Muema, Tarylee Reddy, Yashica Ganga, Farina Karim, Alasdair Leslie, Alex Sigal, Thumbi Ndung’u, the COMMIT-KZN Team

**Affiliations:** 1 HIV Pathogenesis Programme, University of KwaZulu-Natal, Durban, South Africa; 2 Africa Health Research Institute, KwaZulu-Natal, South Africa; 3 Division of Infection and Immunity, University College London, London, UK; 4 School of Laboratory Medicine and Medical Sciences, University of KwaZulu-Natal, Durban, South Africa; 5 Biostatistics Unit, South African Medical Research Council, Durban, South Africa; 6 Max Planck Institute for Infection Biology, Berlin, Germany

**Keywords:** SARS-CoV-2, antibodies, neutralization, South Africa

## Abstract

**Background:**

There is limited understanding of SARS-CoV-2 pathogenesis in African populations with a high burden of infectious disease comorbidities such as HIV. The kinetics, magnitude and duration of virus-specific antibodies and the underlying B cell responses in people living with HIV (PLWH) in sub-Saharan Africa have not been fully characterized.

**Methods:**

We longitudinally followed SARS-CoV-2 infected individuals in Durban, KwaZulu-Natal, South Africa and characterized SARS-CoV-2 receptor binding domain-specific IgM, IgG and IgA antibodies weekly for a month, and then at 3 months post diagnosis. 7/30 (41.7%) were PLWH, 83% (25/30) of which were on ART and with full HIV suppression. Potency of convalescent plasma neutralization was determined using a live virus neutralization assay and antibody secreting cell population frequencies were determined by flow cytometry.

**Results:**

Similar seroconversion rates, time to peak antibody titer, peak magnitude and durability of anti-SARS-CoV-2 IgM, IgG, IgA, were observed in HIV uninfected and PLWH with complete HIV suppression on ART. In addition, similar neutralization potency against an isolate of SARS-CoV-2, circulating at the time of sampling in the first wave of SARS-CoV-2 infections in South Africa was observed in both groups. Loss of IgA was significantly associated with age (p=0.023) and a previous diagnosis of TB (p=0.018).

**Conclusions:**

Similar antibody response kinetics and neutralization potency in HIV negative and PLWH on stable ART in an African setting suggests that COVID-19 natural infections may confer comparable antibody immunity in these groups. This provides hope that COVID-19 vaccines will be effective in PLWH on stable ART.

## Introduction

The COVID-19 pandemic caused by the SARS-CoV-2 virus has significantly impacted global health. Yet, there is relatively limited understanding of its impact in sub-Saharan African populations, with most studies conducted in developed countries. The heterogeneity in severity of COVID-19 has shown the importance of characterization of clinical outcomes and the corresponding immune responses across different populations. This is particularly important for sub-Saharan Africa (sSA) as this region has a higher burden of infectious diseases when compared with other regions. It bears the greatest burden of malaria, tuberculosis and HIV among other endemic infectious diseases that could significantly modulate the immunological profiles of individuals [1]. Indeed, poor immunological responsiveness to Ebola and Yellow Fever vaccines have been reported in African populations when compared to European populations, and this has been attributed to high baseline inflammatory profiles in African populations, even though genetic differences cannot be ruled out [2, 3].

Further, sSA bears the greatest burden of HIV; of the 37.9 million people living with HIV (PLWH) globally, 25.7 million reside in sSA [4]. Some countries in the region have particularly high HIV prevalence, such as South Africa especially KwaZulu-Natal, with a prevalence rate of 19% [5]. Notably, 48.0% of PLWH in sSA remain viremic, suggesting that a large proportion of PLWH in the region could be immunosuppressed. Moreover, antiretroviral therapy reduces but does not fully eliminate HIV-induced inflammation and immune activation, suggesting that some immune defects may persist despite fully suppressive antiretroviral therapy [6, 7]. Population cohort studies report an association of HIV infection and higher disease severity and/or mortality in COVID-19 patients [8-10]. The neutralizing antibody response to SARS-CoV-2 is a key correlate of protection [11], HIV-induced impairment of anti-SARS-CoV-2 neutralizing antibody responses could result in higher disease severity and mortality in COVID-19 patients. The antibody response to SARS-CoV2, in PLWH has, however, not been well characterized to date with most studies focusing on clinical outcome. HIV is known to affect multiple components of the immune system, including the B-cell compartment [12], and PLWH make poor antibody responses to routine vaccination or upon exposure to other natural infections [13-16]. Effective control of HIV viremia with antiretroviral drugs improves responsiveness to routine vaccines, especially when antiretroviral therapy is initiated early in the infection [13, 15, 17]. Considering that anti-SARS-CoV-2 antibodies effectively prevent SARS-CoV-2 infection in COVID-19 convalescent individuals and COVID-19 vaccine recipients, understanding the impact of HIV on their elicitation will reveal possible interactions between the two diseases in sSA. Antibodies are also important diagnostic and surveillance tools [18, 19] and understanding whether there are differences in responses between PLWH and the HIV uninfected population may have implications for the development of diagnostic and surveillance algorithms particularly in regions with high HIV burden.

To characterize the general B-cell response to a live SARS-CoV-2 isolate (B.1.1.117, referred to here as D614G to denote the only mutation of significance) representing a typical SARS-CoV-2 infecting virus in sSA at the time of sampling, we report on the levels of anti-SARS-CoV-2 IgM, IgG, IgA, neutralizing antibodies, and antibody secreting cells (ASC) in a South African cohort of SARS-CoV-2 confirmed cases. We also disaggregate the study population based on HIV status to assess the impact of HIV on anti-SARS-CoV-2 antibody responses in PLWH on antiretroviral treatment (ART).

## Materials and Methods

### Ethical approval

The study protocol was approved by the University of KwaZulu-Natal Institutional Review Board (approval BREC/00001275/2020). Written informed consent was obtained for all participants.

### Participant enrolment

All study participants were over 18 years old, capable of giving informed consent, presented with a positive SARS-CoV-2 polymerase chain reaction (PCR)-based diagnosis, and were recruited from two hospitals (King Edward VIII and Clairwood) in Durban, KwaZulu-Natal, South Africa, between June and November 2020 (last enrolment date was 18 August 2020 after which no participants tested positive for SARS-CoV-2 by PCR indicating that there were no reinfections). Participants consented to blood and nasopharyngeal/oropharyngeal swab collection at recruitment to the study and during weekly follow-up visits until day 28 and a further time point at 3 months post-enrolment. Each participant was subjected to a SARS-CoV-2 RT-qPCR test which also served to quantify the SARS-CoV-2 viral load. Full genome SARS-CoV-2 sequencing was performed for only 16 participants (data not shown). All participants were ranked according to the World Health Organization (WHO) ordinal scale for clinical improvement (https://www.who.int/blueprint/priority-diseases/key-action/COVID-19_Treatment_Trial_Design_Master_Protocol_synopsis_Final_18022020.pdf, accessed February 18, 2020) as (1) ambulatory, no limitation of activities, (2) infected with limitation of activity (3) infected with limitation of activity and hospitalized and (4) infected, hospitalized, and supplemental oxygen provided (Table 1).

**Real Time-qPCR detection of SARS-CoV-2**

The QIAmp Viral RNA Mini kit (QIAGEN, Hilden, Germany) was used according to manufacturer's instructions to extract SARS-CoV-2 RNA. Three SARS-CoV-2 genes (ORF1ab, S and N) were amplified using the TaqPath COVID-19 Combo kit and TaqPath COVID-19 CE-IVD RT-PCR kit (ThermoFisher Scientific, Waltham, MA, USA) using a QuantStudio™7 Flex Real-Time PCR system and analyzed using the Design and Analysis software (ThermoFisher Scientific). Results were interpreted as positive if at least two of the three genes were amplified and regarded inconclusive if only one of the three genes were detected.

**Clinical laboratory testing**

A separate blood sample per participant was sent to an accredited diagnostic laboratory (Molecular Diagnostic Services, Durban, KwaZulu-Natal, South Africa) for HIV testing by rapid test and quantification of HIV viral load. Blood CD4 and CD8 cell counts (cells/µl) were performed by a commercial diagnostic laboratory (Ampath, Durban, South Africa) (Supplementary Table S1).

**Receptor binding domain antibody immune assay**

Plasma samples collected were tested for anti-SARS-CoV-2 IgM, IgG and IgA antibodies as earlier described, but with some modifications. Briefly, flat bottom microplates (ThermoFisher Scientific) were coated with 500 ng/ml of the receptor binding domain (RBD) of the spike protein from SARS-CoV-2 (GenBank: MN975262) (provided by Dr Galit Alter from the Ragon Institute, Cambridge, Massachusetts, USA) and incubated overnight at 4°C. Plates were blocked with a 200 µl/well tris-buffered saline containing 1% BSA (TBSA) and incubated at room temperature (RT) for 1 hour. Samples were diluted in TBSA with 0.05% Tween-20 (TBSAT) to 1:100, 1:1,000 and 1:10,000. Subsequently, goat anti-human IgG (1:5,000), IgM (1:5,000), and IgA (1:10,000)- horseradish peroxidase conjugated secondary antibodies (Jackson ImmunoResearch, West Grove, PA, USA) were added to the respective plates (100 µl/well) and incubated at RT for 1 hour. Bound secondary antibodies were detected using 1-step Ultra TMB substrate (100 µl/well) (ThermoFisher Scientific). Plates were incubated at RT for 3 (IgG) and 5 (IgM and IgA) min respectively in the dark before addition of 1 N sulphuric acid stop solution (100 µl/well). Plates were washed with high salt TBS containing 0.05% Tween-20 after each incubation. Standard curves were used to calculate the concentration (ng/mL) of anti-RBD expressed as IgG (anti-SARS-CoV-2 monoclonal, CR3022), IgM (anti-SARS-CoV-2 S1 RBD IgM, hIgM2001) or IgA (anti-SARS-CoV-2 S1 RBD IgA, hIgA2001) (Genscript Piscataway, NJ, USA) [20, 21]. We used pre-pandemic plasma samples as negative controls to define seroconversion cut-offs calculated as three times the standard deviation plus the mean of the negative samples for each of the isotypes.

**Live virus micro- neutralization assay (LVNA)**

A live virus neutralization focus-forming assay (LVNA) using an isolate of SARS-CoV-2 (D614G) [22] with the following modifications: input virus was 100 focus-forming units (FFU) per well and plasma samples were serially diluted four-fold from 1:20 to 1:81,920 final concentrations. For each participant, one early (median days post-symptom onset: 30 (IQR: 26-62)) and one late (median 102 (IQR: 98-111) time point was selected. The dilution of sera required to inhibit viral infection by 50% relative to the no-antibody control wells (NT50) was calculated using a four-parameter logistic curve fit (GraphPad Prism version 9.01).

**Quantification of antibody-secreting cells**

Blood was collected in EDTA tubes and diluted 1:3 with PBS. Peripheral blood mononuclear cells (PBMC) were isolated by density gradient centrifugation through Histopaque 1077 (Sigma-Aldrich) in SepMate separation tubes (STEMCELL Technologies, Vancouver,

Canada). For immune phenotyping $10^6$ fresh PBMC were surface stained in a 25 µl antibody mix containing a LIVE/DEAD™ fixable near-IR-dead cell staining reagent (1:200 dilution, Invitrogen, Carlsbad, CA, USA) with combinations of the listed antibodies from BD Biosciences (Franklin Lakes, NJ, USA) or from BioLegend (San Diego, CA, USA) or from Beckman Coulter (Brea, CA, USA). Cells were stained for 20 min in the dark at RT, followed by two 1 ml washes with cold PBS, then fixed in 2% paraformaldehyde and stored at 4°C until acquisition on a FACSAria Fusion III flow cytometer (BD). CD19+ B cells were analyzed for the B cell maturation markers CD27 and CD38, to identify antibody secreting cell (ASC, CD27+CD38++) using FlowJo version 9.9.6 (Tree Star).

**Statistical analysis**

Statistical analyses were conducted in Stata (version 16) and GraphPad Prism (version 9.01). Lowess curves were generated to indicate kinetics of antibodies over time. Kaplan Meier curves (multivariable analyses) were used to determine time to seroconversion and Pearson's chi square test to determine factors associated with the loss of antibodies, with 95% Confidence intervals (CI). Cox regression and Breslow method for ties, were used to determine any associations to antibody responses. Spearman's rank test was used to determine all correlations. P<0.05 were considered significant.

## Results

A total of 72 SARS-CoV-2 infected patients with 294 clinical specimens were analyzed in this longitudinal study. Full genome SARS-CoV-2 sequence data was available for 16 participants only none of which was infected with a variant of concern (data not shown). Most participants were women (55/72, 76.4%), the median age was 43.4 (IQR:33.3-51.9), 30/72 (41.7%) were HIV positive, of which 25/30 (83.0%) were on ART and fully HIV suppressed in the blood. CD4 cell counts were significantly lower (p=0.0006) in PLWH

while CD8 cell counts were significantly higher (p=0.0008) PLWH. Hypertension was the leading co-morbidity (19.4%) in participants followed by diabetes (18.1%) and having a history of TB were significantly more common in PLWH (p=0.008). Of the participants, 6/72 (12.5%) had no clinical manifestation, 45/72 (62.5%) had mild clinical symptoms with limitation of activities, and 18/72 (25.0%) had severe clinical symptoms and required supplemental oxygen (Table 1). For all analyses, days from symptom onset were used or day of enrolment for asymptomatic cases.

Anti-SARS-CoV-2 IgM, IgG, and IgA antibodies specific to the RBD were measured for all participants, sampled weekly up to 28 days with a further time point at 3 months (Figure 1a-c). Overall, 67/72 (93.1%) participants had more than one measurement within 28 days and the majority possessing SARS-CoV2 specific IgM and IgG antibodies (53/72, 73.6% and 70/72 97.2%, respectively). SARS-CoV2 IgA antibodies were less frequently observed, but still present in most individuals (43/72, 59.7%) (Figure 1a-c). Samples were available for 43/72 (59.7%) participants at the 3 months timepoint post enrolment. These samples showed a marked waning of the IgM (8/43;18.6%) and IgA responses (17/43; 39.5%), but IgG were well maintained (37/43; 86.1%). Using the cut-offs defined earlier, we estimated the distribution of the time required to seroconversion for IgM, IgG, and IgA (Figure 1a-c). The estimated median time to seroconversion for IgM was 17 days (IQR: 13–19), IgG 13 days (IQR: 11–16) and IgA 19 days (IQR: 16–31). Loss of IgA antibodies was significantly associated with age (p=0.023) and having a history of TB (p=0.0018) while no trend could be seen for IgM or IgG.

Antibody responses were next stratified by HIV status and overall, no differences in maximal anti-SARS-CoV-2 IgM, IgG, or IgA titers were observed (Figure 1d-f). Early and acute antibody responses were similar between HIV negative and PLWH with significantly higher responses during the convalescent weeks in PLWH (weeks 8, 7 and 7 for IgM

(p=0.0455), IgG (p=0.0133), and IgA (p=0.0133) respectively) (Supplementary Figure S1). There was also no difference in time to seroconversion between PLWH and HIV negative participants for any isotype. HIV-associated parameters (HIV plasma viral load, CD4, CD8 cell counts and CD4:CD8 ratio) were also not associated with significant antibody losses in PLWH across all three isotypes. A history of TB (only 6 participants with a history of TB developed IgA) (p=0.0018) and age (34-45 years) (p=0.023) were associated with loss of IgA antibodies at 3-month post symptom onset (Figure 2a, b) while no trend was noted for IgM or IgG.

Using a live virus focus-forming assays to measure anti-SARS-CoV-2 neutralization (NT50) we observed no difference in neutralizing potency between PLWH and HIV negative participants (Figure 3a). Anti-SARS-CoV-2 neutralization positively correlated most strongly with IgG titers from the same timepoints ($r_s$=0.9, p<0.001), followed by IgA ($r_s$=0.7, p<0.001) and IgM ($r_s$=0.5, p<0.001) (Figure 3b). No significant differences in the neutralization ability were detected between PLHW and HIV uninfected participants. To examine the cellular correlates of neutralizing antibodies, we measured the frequency of CD27+CD38++ ASC at baseline, in a subset of individuals by flow cytometry (Figure 4a-d). ASC, measured at 0–13 days post symptom onset, significantly correlated with IgM ($r_s$=0.66, p=0.03) and IgA ($r_s$=0.71, p=0.02) titers with a similar correlation observed for IgG ($r_s$=0.56, p=0.07) and neutralizing ($r_s$=0.56, p=0.09) antibodies measured at 35–68 days post symptom onset.

Finally, we tested the relationship between SARS-CoV-2 specific antibodies and clinical parameters by regression analysis. Anti-SARS-CoV-2 IgM antibody responses were significantly associated with disease severity and the need for supplemental oxygen (HR: IgM 6.3 (95%CI: 1.4–27.4), p=0.015) (Figure 5a). IgA antibody production was significantly associated with older age (45–54 years; HR 2.9 (95%CI: 1.1–7.2), p=0.02; >55 years; HR:

2.9 (95%CI: 1.2–7.3), p=0.02) and hypertension (HR: 2.3 (95%CI: 1.2–4.4), p=0.01) (Figure 5c). IgG antibody production was not associated with any clinical co-variates. Strikingly, and in line with the data presented, HIV co-infection (negative versus positive), HIV viral load (uninfected versus HIV virally suppressed and HIV viremic), CD4 and CD8 cell counts were not associated with differences in any of the antibody isotypes measured (Figure 5).

**Discussion**

This study reports on longitudinal anti-SARS-CoV-2 IgM, IgG and IgA antibody titers and neutralization activity in a sub-Saharan African population with a high burden of HIV. There was no difference in IgM, IgG or IgA kinetics, durability, or neutralization potency between HIV uninfected participants and PLWH, 83% of whom were fully HIV suppressed. These data agree with recently published data who showed similar seroconversion kinetics in PLWH with well-controlled disease and HIV uninfected individuals [23-26] highlighting the importance of a secure supply of ART for PLWH during the COVID-19 pandemic. Overall, a lack of individuals with unsuppressed HIV infection were available to determine its effect of uncontrolled HIV in SARS-CoV2 serology. Nonetheless, these data demonstrate that HIV co-infection per se does not appear to limit the initial antibody response in the first SARS-CoV-2 infection wave in South Africa. Numerous studies have demonstrated the effect of ART on vaccine-modulated immunity against polio and measles. As such, a similar benefit of ART could lead to the observed similarities between HIV-positive and HIV-negative individuals in this study.

Overall, age was significantly associated with both production and loss of IgA. This trend, however, was not observed when stratified by HIV status, probably a limitation of the sample size. Interestingly, loss of IgA was also associated with a previous diagnosis of TB. Current and previous diagnoses of TB have also been associated with COVID-19 death in a population cohort study in South Africa [9]. In other studies, active TB disease has been associated with a lower frequency of B cells and a higher frequency of atypical double-negative B cells; and whereas the frequency of total B cells normalized after treatment, individuals with a history of TB treatment still had higher double-negative B cells [27]. Therefore, active TB might affect the humoral response to other pathogens including SARS-CoV-2. Since *Mycobacterium tuberculosis* and SARS-CoV-2 are both respiratory pathogens, existing TB-related damage to the mucous and respiratory membranes could also be further exacerbated by SARS-CoV-2 infection and affect mucosal immunity. These potential interactions between respiratory pathogens need further investigation. Antibody secreting cells at 0-13 days post symptom onset positively correlated with IgG and IgA production 35-68 days post symptom onset. Due to the small sample size, we could not stratify into PLWH and HIV negative groups. A recent study, however, reported no difference in ASC between these two groups [28].

Multiple studies have shown that most SARS-CoV-2 infected individuals produce S- and RBD-specific antibodies during the primary response, and RBD-specific monoclonal antibodies can neutralize the virus *in vitro* and *in vivo [29]*. A recent study had demonstrated that higher sensitivity of the S protein versus the nucleocapsid protein for both acute and post-infection phase with the anti-N IgG antibodies waning after acute infection [30]. Therefore, RBD-specific antibodies would likely contribute to protection against re-infection. These results are similar to previous reports. Whether the neutralization response is also similar in subsequent infection waves with variants that show multiple functional differences to the original circulating strains is yet to be determined.

Limitations to the study include the following 1) the small sample size of HIV viremic participants. Only five people were HIV viremic and we can therefore only conclude on the effect of HIV on antibody kinetics in ART virally suppressed individuals, as well as on the impact that HIV-associated parameters such as CD4 cell counts have on antibody responses. Future studies should focus on HIV suppressed versus HIV viremic patients; 2) base-line blood at enrollment were taken between 0–37 days post symptom onset. Consequently, we may have missed the peak IgM response in some individuals and time to seroconversion may be shorter than reported here; 3) Only 16/72 SARS-CoV-2 full genomes were available and therefore we are unable to confirm the infecting viral variants. However, it should be noted that all participants were enrolled into the study by mid-August 2020, prior to the circulation of the Beta VOC; 4) The association between loss of IgA antibodies, TB and age should be interpreted with caution due to the small sample size.

Despite these caveats, these data show similar antibody kinetics, durability, and neutralization potency in PLWH on stable ART and HIV negative people for SARS-CoV-2 D614G during the first wave of infections in an African setting. This provides hope that COVID-19 vaccines will be effective in PLWH on stable ART.

Accepted M

NOTES

**Acknowledgments**

We thank the study participants, their clinicians, and members of the collaborating teams in the COMMIT-KZN consortium.

**Funding**

This work was supported by a grant from the Wellcome Trust to the Africa Health Research Institute (Wellcome Trust Strategic Core Award [grant number 201433/Z/16/Z]). Additional funding was received from the Bill and Melinda Gates Foundation [grant number INV-018944], the South African Department of Science and Innovation through the National Research Foundation (South African Research Chairs Initiative, [grant number 64809]), and the Victor Daitz Foundation. Additional funding was provided by the Sub-Saharan African Network for TB/HIV Research Excellence (SANTHE), a DELTAS Africa Initiative [grant number DEL-15-006]. The DELTAS Africa Initiative is an independent funding scheme of the African Academy of Sciences (AAS)'s Alliance for Accelerating Excellence in Science in Africa (AESA) and supported by the New Partnership for Africa's Development Planning and Coordinating Agency (NEPAD Agency) with funding from the Wellcome Trust [grant number 107752/Z/15/Z] and the UK government. The views expressed in this publication are those of the author(s) and not necessarily those of AAS, NEPAD Agency, Wellcome Trust, or the UK government.

**Declaration of interests**

The authors declare no competing interests.

**Commit KZN Team**

Moherndran Archary[1], Kaylesh J. Dullabh[2], Philip Goulder[3,4], Guy Harling[3,5], Rohen Harrichandparsad[6], Kobus Herbst[3,7], Prakash Jeena[1], Thandeka Khoza[3], Nigel Klein[3,8], Henrik Kløverpris[3,9,10], Rajhmun Madansein[2], Mohlopheni Marakalala[3,9], Matilda Mazibuko[3], Mosa Moshabela[11], Ntombifuthi Mthabela[3], Kogie Naidoo[12], Zaza Ndhlovu[3,13], Kennedy Nyamande[14], Nesri Padayatchi[12], Vinod Patel[15], Theresa Smit[3], Adrie Steyn[3,16], Emily Wong[3,16].

[1]Department of Paediatrics and Child Health, University of KwaZulu-Natal, Durban, South Africa. [2]Department of Cardiothoracic Surgery, University of KwaZulu-Natal, Durban, South Africa. [3]Africa Health Research Institute, Durban, South Africa. [4]Department of Paediatrics, Oxford, UK. [5]Institute for Global Health, University College London, UK. 6Department of Neurosurgery, University of KwaZulu-Natal, Durban, South Africa. [7]South African Population Research Infrastructure Network, Durban, South Africa. [8]Institute of Child Health, University College London, UK. [9]Division of Infection and Immunity, University College London, London, UK.[10]Department of Immunology and Microbiology, University of Copenhagen, Copenhagen, Denmark. [11]College of Health Sciences, University of KwaZulu-Natal, Durban, South Africa. [12]Centre for the AIDS Programme of Research in South Africa, Durban, South Africa. [13]Ragon Institute of MGH, MIT and Harvard, Boston, USA. [14]Department of Pulmonology and Critical Care, University of KwaZulu-Natal, Durban, South Africa.[15]Department of Neurology, University of KwaZulu-Natal, Durban, South Africa. [16]Division of Infectious Diseases, University of Alabama at Birmingham.

## References

[1] GBD. Global Burden of Diseases. 2021(Accessed on 19th May 2021):https://vizhub.healthdata.org/gbd-compare/.

[2] Pasin C, Balelli I, Van Effelterre T, Bockstal V, Solforosi L, Prague M, et al. Dynamics of the Humoral Immune Response to a Prime-Boost Ebola Vaccine: Quantification and Sources of Variation. Journal of virology 2019;93(18):e00579-19.

[3] Muyanja E, Ssemaganda A, Ngauv P, Cubas R, Perrin H, Srinivasan D, et al. Immune activation alters cellular and humoral responses to yellow fever 17D vaccine. J Clin Invest 2014;124(7):3147-58.

[4] WHO. HIV/AIDS: Disease burden. 2021(Accessed on 19th May 2021):https://www.afro.who.int/health-topics/hivaids.

[5] UNAIDS. Country factsheets: South Africa-2019. 2021(Accessed on 19th May 2021):https://www.unaids.org/en/regionscountries/countries/southafrica.

[6] Nabatanzi R, Bayigga L, Cose S, Rowland Jones S, Joloba M, Canderan G, et al. Monocyte Dysfunction, Activation, and Inflammation After Long-Term Antiretroviral Therapy in an African Cohort. J Infect Dis 2019;220(9):1414-9.

[7] Wada NI, Jacobson LP, Margolick JB, Breen EC, Macatangay B, Penugonda S, et al. The effect of HAART-induced HIV suppression on circulating markers of inflammation and immune activation. AIDS (London, England) 2015;29(4):463-71.

[8] Bhaskaran K, Rentsch CT, MacKenna B, Schultze A, Mehrkar A, Bates CJ, et al. HIV infection and COVID-19 death: a population-based cohort analysis of UK primary care data and linked national death registrations within the OpenSAFELY platform. Lancet HIV 2021;8(1):e24-e32.

[9] Boulle A, Davies MA, Hussey H, Ismail M, Morden E, Vundle Z, et al. Risk factors for COVID-19 death in a population cohort study from the Western Cape Province, South Africa. Clin Infect Dis 2020.

[10] Tesoriero JM, Swain CE, Pierce JL, Zamboni L, Wu M, Holtgrave DR, et al. COVID-19 Outcomes Among Persons Living With or Without Diagnosed HIV Infection in New York State. JAMA Netw Open 2021;4(2):e2037069.

[11] Khoury DS, Cromer D, Reynaldi A, Schlub TE, Wheatley AK, Juno JA, et al. Neutralizing antibody levels are highly predictive of immune protection from symptomatic SARS-CoV-2 infection. Nature Medicine 2021.

[12] Lane HC, Masur H, Edgar LC, Whalen G, Rook AH, Fauci AS. Abnormalities of B-cell activation and immunoregulation in patients with the acquired immunodeficiency syndrome. N Engl J Med 1983;309(8):453-8.

[13] Pensieroso S, Cagigi A, Palma P, Nilsson A, Capponi C, Freda E, et al. Timing of HAART defines the integrity of memory B cells and the longevity of humoral responses in HIV-1 vertically-infected children. Proc Natl Acad Sci U S A 2009;106(19):7939-44.

[14] Helfand RF, Witte D, Fowlkes A, Garcia P, Yang C, Fudzulani R, et al. Evaluation of the immune response to a 2-dose measles vaccination schedule administered at 6 and 9 months of age to HIV-infected and HIV-uninfected children in Malawi. The Journal of infectious diseases 2008;198(10):1457-65.

[15] Moir S, Buckner CM, Ho J, Wang W, Chen J, Waldner AJ, et al. B cells in early and chronic HIV infection: evidence for preservation of immune function associated with early initiation of antiretroviral therapy. Blood 2010;116(25):5571-9.

[16] Muema DM, Macharia GN, Hassan AS, Mwaringa SM, Fegan GW, Berkley JA, et al. Control of Viremia Enables Acquisition of Resting Memory B Cells with Age and

Normalization of Activated B Cell Phenotypes in HIV-Infected Children. J Immunol 2015;195(3):1082-91.

[17] Gelinck LB, Jol-van der Zijde CM, Jansen-Hoogendijk AM, Brinkman DM, van Dissel JT, van Tol MJ, et al. Restoration of the antibody response upon rabies vaccination in HIV-infected patients treated with HAART. AIDS 2009;23(18):2451-8.

[18] Fenwick C, Croxatto A, Coste AT, Pojer F, André C, Pellaton C, et al. Changes in SARS-CoV-2 Spike versus Nucleoprotein Antibody Responses Impact the Estimates of Infections in Population-Based Seroprevalence Studies. Journal of virology 2021;95(3).

[19] Uyoga S, Adetifa IMO, Karanja HK, Nyagwange J, Tuju J, Wanjiku P, et al. Seroprevalence of anti–SARS-CoV-2 IgG antibodies in Kenyan blood donors. Science 2021;371(6524):79-82.

[20] Iyer AS, Jones FK, Nodoushani A, Kelly M, Becker M, Slater D, et al. Persistence and decay of human antibody responses to the receptor binding domain of SARS-CoV-2 spike protein in COVID-19 patients. Science Immunology 2020;5(52):eabe0367.

[21] Wang C, Li W, Drabek D, Okba NMA, van Haperen R, Osterhaus A, et al. Publisher Correction: A human monoclonal antibody blocking SARS-CoV-2 infection. Nature communications 2020;11(1):2511.

[22] Cele S, Gazy I, Jackson L, Hwa S-H, Tegally H, Lustig G, et al. Escape of SARS-CoV-2 501Y.V2 from neutralization by convalescent plasma. medRxiv 2021:2021.01.26.21250224.

[23] Yamamoto S, Saito M, Nagai E, Toriuchi K, Nagai H, Yotsuyanagi H, et al. Antibody response to SARS-CoV-2 in people living with HIV. J Microbiol Immunol Infect 2021;54(1):144-6.

[24] Pallikkuth S, Sharkey M, Beauchamps L, Raccamarich P, Uribe C, Salazar A, et al. SARS-COV-2–specific AB response in HIV+ individuals on ART. *Virtual Conference on Retroviruses and Opportunistic Infections.* San Francisco, CA; 2021.

[25] Alrubayyi A, Gea-Mallorqui E, Touizer E, Bowen D, Kopycinski J, Charlton B, et al. Characterization of SARS-CoV-2–specific responses in people living with HIV. *Virtual Conference on Retroviruses and Opportunistic Infections.* San Francisco, CA; 2021.

[26] Cooper TJ, Woodward BL, Alom S, Harky A. Coronavirus disease 2019 (COVID-19) outcomes in HIV/AIDS patients: a systematic review. HIV Med 2020;21(9):567-77.

[27] Joosten SA, van Meijgaarden KE, del Nonno F, Baiocchini A, Petrone L, Vanini V, et al. Patients with Tuberculosis Have a Dysfunctional Circulating B-Cell Compartment, Which Normalizes following Successful Treatment. PLoS pathogens 2016;12(6):e1005687.

[28] Karim F, Gazy I, Cele S, Zungu Y, Krause R, Bernstein M, et al. HIV infection alters SARS-CoV-2 responsive immune parameters but not clinical outcomes in COVID-19 disease. medRxiv 2020:2020.11.23.20236828.

[29] Shi R, Shan C, Duan X, Chen Z, Liu P, Song J, et al. A human neutralizing antibody targets the receptor-binding site of SARS-CoV-2. Nature 2020;584(7819):120-4.

[30] Fenwick C, Croxatto A, Coste AT, Pojer F, André C, Pellaton C, et al. Changes in SARS-CoV-2 Spike versus Nucleoprotein Antibody Responses Impact the Estimates of Infections in Population-Based Seroprevalence Studies. Journal of virology 2020:JVI.01828-20.

## Figure legends

**Figure 1:** Effect of HIV status on temporal responses of different SARS-CoV-2 specific antibody isotypes. (a-c) IgM, IgG, and IgA of all 72 participants (295 timepoints total) measured weekly up to 28 days and at 3-months post symptom inset. (d-f) Stratified according to PLWH (indicated in red) and HIV negative (indicated in blue). Locally weighted scatterplot smoothing curves were used to fit the data. Cut-offs for seroconversion are indicated on the y-axis with a dotted line at 3 $\log_{10}$ for IgM and IgG and 2.6 $\log_{10}$ for IgA.

**Figure 2:** Loss of anti-SARS-CoV-2 IgA antibodies at 3-months post symptom onset, was significantly associated with having a) a history of TB (n=6, p=0.0018) as well as age b) age (n=43, p=0.023).

**Figure 3:** Neutralization by convalescent plasma of D614G SARS-CoV-2 using a live virus neutralization assay. a) Neutralization measured as NT50 in HIV negative participants (blue) and PLWH (red). The horizontal line indicates the most concentrated plasma dilution tested in the assay. Shown is median and interquartile range for n=59 HIV negative participants and n=40 PLWH. b) Correlation between SARS-CoV-2 anti-IgG and IgA binding- and neutralization capacity.

**Figure 4:** Correlation between frequency of antibody secreting cells and antibody isotypes. Concentrations of anti-SARS-CoV2 a) IgM, b) IgG, c) IgA and d) neutralizing antibody production 35-68 days post-symptom onset as a function of the frequency of antibody secreting cells (ASC) 0-13 days post-symptom onset.

**Figure 5:** Hazard Ratios for covariates which impact anti-SARS-CoV-2 antibody production. Hazard ratios and 95% confidence intervals for a) IgM, b) IgG and c) IgA with significant associations indicated at $p<0.05$. $^{*}$HIV virally suppressed and HIV viremic participants are compared to HIV uninfected participants; $^{\Psi}$Association of antibody responses with CD4 and CD8 cell counts in HIV positive participants; $^{\Pi}$Association of age groups with antibody responses (participants <35 years are the reference group); $^{\varpi}$Association of antibody responses with disease severity (asymptomatic participants (disease severity score 1 are the reference group).

**Table 1: Participant enrolment information**

| n, % | All (n=72) | HIV- (n=42, 58.3%) | HIV+ (n=30, 41.7%) | p-value (stratified by HIV status) |
|---|---|---|---|---|
| Females | 55 (76.4%) | 32 (76.2%) | 23 (76.7%) | 0.9 |
| Age years, median (IQR) | 43.8 (33.3-51.9) | 45.1 (32.5-54.9) | 42.8 (34.9-50.5) | 0.5 |
| **Disease severity** (ordinal scale*) | | | | 0.883 |
| Asymptomatic (1) | 9 (12.5%) | 6 (14.3%) | 3 (10.0%) | |
| Mild (2/3) | 45 (62.5%) | 26 (61.9%) | 19 (63.3%) | |
| Severe (4) | 18 (25.0%) | 10 (23.8%) | 8 (26.7%) | |
| **Co-morbidities** | | | | |
| Hypertension | 14 (19.4%) | 9 (21.4%) | 5 (16.7%) | 0.765 |
| Diabetes | 13 (18.1%) | 10 (23.8%) | 3 (10.0%) | 0.214 |
| History of TB | 8 (11.1%) | 1 (2.4%) | 7 (23.3%) | 0.008 |

*World Health Organization ordinal scale for clinical improvement (https://www.who.int/blueprint/priority-diseases/key-action/COVID-19_Treatment_Trial_Design_Master_Protocol_synopsis_Final_18022020.pdf, February 18, 2020)

Figure 1

a)
b)
c)
d)
e)
f)
Log10 IgM (ng/ml)
Log10 IgG (ng/ml)
Log10 IgA (ng/ml)
Pre-pandemic controls
Days post symptom onset
HIV-
HIV+

Figure 2

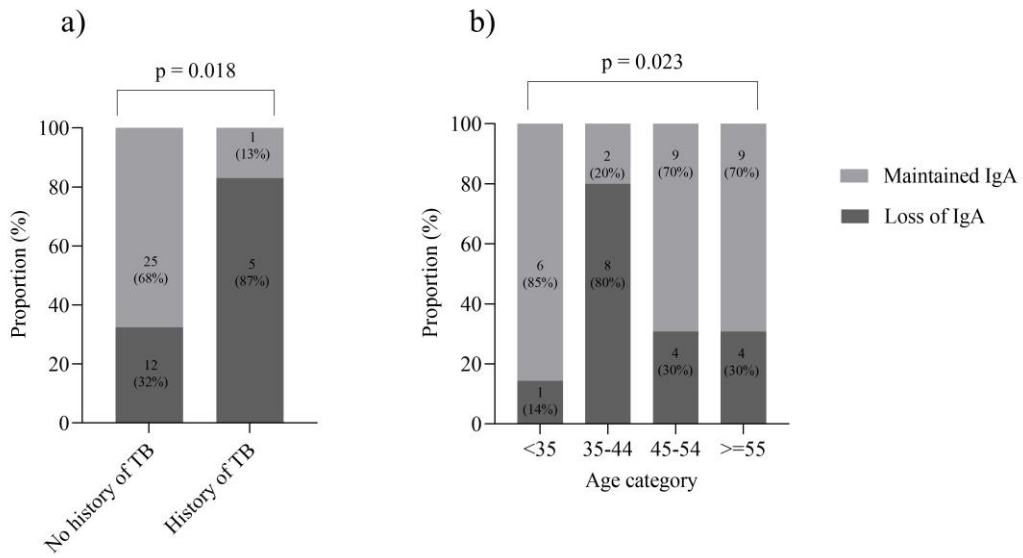

Figure 3

a)
ns
Log10(NT50)
LOQ
HIV-
HIV+
b)
IgA IgM/IgG
IgM rs=0.5, p<0.0001
IgG rs=0.9, p<0.0001
IgA rs=0.7, p<0.0001
Log10(NT50)
LOQ
Log10 antibody concentration (ng/ml)

Figure 4

Figure 5

## Supplementary Material

ciab758_suppl_Supplementary_Data

