# Similar Antibody Responses Against Severe Acute Respiratory Syndrome Coronavirus 2 in Individuals Living Without and With Human Immunodeficiency Virus on Antiretroviral Therapy During the First South African Infection Wave

**DOI:** 10.1093/cid/ciab758

**Published:** 2021-09-02

**Authors:** Jumari Snyman, Shi Hsia Hwa, Robert Krause, Daniel Muema, Tarylee Reddy, Yashica Ganga, Farina Karim, Alasdair Leslie, Alex Sigal, Thumbi Ndung’u, COVID-19 Mechanisms and Multi-omics at the Intersection of TB and HIV in KwaZulu-Natal (COMMIT-KZN Team), Moherndran Archary, Kaylesh J Dullabh, Philip Goulder, Guy Harling, Rohen Harrichandparsad, Kobus Herbst, Prakash Jeena, Thandeka Khoza, Nigel Klein, Henrik Kløverpris, Rajhmun Madansein, Mohlopheni Marakalala, Matilda Mazibuko, Mosa Moshabela, Ntombifuthi Mthabela, Kogie Naidoo, Zaza Ndhlovu, Kennedy Nyamande, Nesri Padayatchi, Vinod Patel, Theresa Smit, Adrie Steyn, Emily Wong

**Affiliations:** HIV Pathogenesis Programme, University of KwaZulu-Natal, Durban, South Africa; Department of Basic and Translational Science, Africa Health Research Institute, KwaZulu-Natal, South Africa; School of Laboratory Medicine and Medical Sciences, University of KwaZulu-Natal, Durban, South Africa; Department of Basic and Translational Science, Africa Health Research Institute, KwaZulu-Natal, South Africa; Division of Infection and Immunity, University College London, London, United Kingdom; Department of Basic and Translational Science, Africa Health Research Institute, KwaZulu-Natal, South Africa; School of Laboratory Medicine and Medical Sciences, University of KwaZulu-Natal, Durban, South Africa; HIV Pathogenesis Programme, University of KwaZulu-Natal, Durban, South Africa; Department of Basic and Translational Science, Africa Health Research Institute, KwaZulu-Natal, South Africa; School of Laboratory Medicine and Medical Sciences, University of KwaZulu-Natal, Durban, South Africa; Biostatistics Unit, South African Medical Research Council, Durban, South Africa; Department of Basic and Translational Science, Africa Health Research Institute, KwaZulu-Natal, South Africa; Department of Basic and Translational Science, Africa Health Research Institute, KwaZulu-Natal, South Africa; School of Laboratory Medicine and Medical Sciences, University of KwaZulu-Natal, Durban, South Africa; Department of Basic and Translational Science, Africa Health Research Institute, KwaZulu-Natal, South Africa; Division of Infection and Immunity, University College London, London, United Kingdom; Department of Basic and Translational Science, Africa Health Research Institute, KwaZulu-Natal, South Africa; School of Laboratory Medicine and Medical Sciences, University of KwaZulu-Natal, Durban, South Africa; Systems Infection Biology Group, Max Planck Institute for Infection Biology, Berlin, Germany; HIV Pathogenesis Programme, University of KwaZulu-Natal, Durban, South Africa; Department of Basic and Translational Science, Africa Health Research Institute, KwaZulu-Natal, South Africa; School of Laboratory Medicine and Medical Sciences, University of KwaZulu-Natal, Durban, South Africa; Division of Infection and Immunity, University College London, London, United Kingdom; Systems Infection Biology Group, Max Planck Institute for Infection Biology, Berlin, Germany; Department of Paediatrics and Child Health, University of KwaZulu-Natal, Durban, South Africa; Department of Cardiothoracic Surgery, University of KwaZulu-Natal, Durban, South Africa; Africa Health Research Institute, Durban, South Africa; Department of Paediatrics, Oxford, United Kingdom; Africa Health Research Institute, Durban, South Africa; Institute for Global Health, University College London, United Kingdom; Department of Neurosurgery, University of KwaZulu-Natal, Durban, South Africa; Africa Health Research Institute, Durban, South Africa; South African Population Research Infrastructure Network, Durban, South Africa; Department of Paediatrics and Child Health, University of KwaZulu-Natal, Durban, South Africa; Africa Health Research Institute, Durban, South Africa; Africa Health Research Institute, Durban, South Africa; Institute of Child Health, University College London, United Kingdom; Africa Health Research Institute, Durban, South Africa; Division of Infection and Immunity, University College London, London, United Kingdom; Department of Immunology and Microbiology, University of Copenhagen, Copenhagen, Denmark; Department of Cardiothoracic Surgery, University of KwaZulu-Natal, Durban, South Africa; Africa Health Research Institute, Durban, South Africa; Division of Infection and Immunity, University College London, London, United Kingdom; Africa Health Research Institute, Durban, South Africa; College of Health Sciences, University of KwaZulu-Natal, Durban, South Africa; Africa Health Research Institute, Durban, South Africa; Centre for the AIDS Programme of Research in South Africa, Durban, South Africa; Africa Health Research Institute, Durban, South Africa; Ragon Institute of Massachusetts General Hospital (MGH), Massachusetts Institute of Technology (MIT) and Harvard, Boston, Massachusetts, USA; Department of Pulmonology and Critical Care, University of KwaZulu-Natal, Durban, South Africa; Centre for the AIDS Programme of Research in South Africa, Durban, South Africa; Department of Neurology, University of KwaZulu-Natal, Durban, South Africa; Africa Health Research Institute, Durban, South Africa; Africa Health Research Institute, Durban, South Africa; Division of Infectious Diseases, University of Alabama–Birmingham, Birmingham, Alabama, USA; Africa Health Research Institute, Durban, South Africa; Division of Infectious Diseases, University of Alabama–Birmingham, Birmingham, Alabama, USA

**Keywords:** SARS-CoV-2, antibodies, neutralization, South Africa

## Abstract

**Background:**

There is limited understanding of severe acute respiratory syndrome coronavirus 2 (SARS-CoV-2) pathogenesis in African populations with a high burden of infectious disease comorbidities such as human immunodeficiency virus (HIV). The kinetics, magnitude, and duration of virus-specific antibodies and B-cell responses in people living with HIV (PLWH) in sub-Saharan Africa have not been fully characterized.

**Methods:**

We longitudinally followed SARS-CoV-2–infected individuals in Durban, KwaZulu-Natal, South Africa, and characterized SARS-CoV-2 receptor-binding domain-specific immunoglobulin (Ig) M, IgG, and IgA weekly for 1 month and at 3 months post-diagnosis. Thirty of 72 (41.7%) were PLWH, 25/30 (83%) of whom were on antiretroviral therapy (ART) with full HIV suppression. Plasma neutralization was determined using a live virus neutralization assay, and antibody-secreting cell population frequencies were determined by flow cytometry.

**Results:**

Similar seroconversion rates, time to peak antibody titer, peak magnitude, and durability of anti–SARS-CoV-2 IgM, IgG, and IgA were observed in people not living with HIV and PLWH with complete HIV suppression on ART. In addition, similar potency in a live virus neutralization assay was observed in both groups. Loss of IgA was significantly associated with age (P = .023) and a previous diagnosis of tuberculosis (*P* = .018).

**Conclusions:**

Similar antibody responses and neutralization potency in people not living with HIV and PLWH on stable ART in an African setting suggest that coronavirus disease 2019 (COVID-19) natural infections may confer comparable antibody immunity in these groups. This provides hope that COVID-19 vaccines will be effective in PLWH on stable ART.

## Teaser

In a longitudinal study during the first COVID-19 infection wave in South Africa, we report no differences in anti-SARS-CoV-2 immunoglobulin M (IgM), IgG, and IgA antibody kinetics, peak titers, and neutralization in people living with HIV versus people without HIV.

The coronavirus disease 2019 (COVID-19) pandemic caused by the severe acute respiratory syndrome coronavirus 2 (SARS-CoV-2) virus has significantly impacted global health. Yet, there is relatively limited understanding of its impact in sub-Saharan African populations, with most studies conducted in developed countries. The heterogeneity in severity of COVID-19 has shown the importance of characterization of clinical outcomes and the corresponding immune responses across different populations. This is particularly important for sub-Saharan Africa as this region has a higher burden of infectious diseases when compared with other regions. It bears the greatest burden of malaria, tuberculosis (TB), and human immunodeficiency virus (HIV) among other endemic infectious diseases that could significantly modulate the immunological profiles of individuals [1]. Indeed, poor immunological responsiveness to Ebola and yellow fever vaccines have been reported in African populations when compared with European populations, and this has been attributed to high baseline inflammatory profiles in African populations, even though genetic differences cannot be ruled out [2, 3].

Further, sub-Saharan Africa bears the greatest burden of HIV; of the 37.9 million people living with HIV (PLWH) globally, 25.7 million reside in sub-Saharan Africa [4]. Some countries in the region have particularly high HIV prevalence, such as South Africa especially KwaZulu-Natal, with a prevalence rate of 19% [5]. Notably, 48.0% of PLWH in sub-Saharan Africa remain viremic, suggesting that a large proportion of PLWH in the region could be immunosuppressed. Moreover, antiretroviral therapy (ART) reduces but does not fully eliminate HIV-induced inflammation and immune activation, suggesting that some immune defects may persist despite fully suppressive ART [6, 7]. Population cohort studies report an association of HIV infection and higher disease severity and/or mortality in COVID-19 patients [8–10]. The neutralizing antibody response to SARS-CoV-2 is a key correlate of protection [11]; HIV-induced impairment of anti–SARS-CoV-2 neutralizing antibody responses could result in higher disease severity and mortality in COVID-19 patients. The antibody response to SARS-CoV-2 in PLWH has, however, not been well characterized to date, with most studies focusing on clinical outcome. HIV is known to affect multiple components of the immune system, including the B-cell compartment [12], and PLWH make poor antibody responses to routine vaccination or on exposure to other natural infections [13–16]. Effective control of HIV viremia with antiretroviral drugs improves responsiveness to routine vaccines, especially when ART is initiated early in the infection [13, 15, 17]. Considering that anti–SARS-CoV-2 antibodies effectively prevent SARS-CoV-2 infection in COVID-19 convalescent individuals and COVID-19 vaccine recipients, understanding the impact of HIV on their elicitation will reveal possible interactions between the 2 diseases in sub-Saharan Africa. Antibodies are also important diagnostic and surveillance tools [18, 19], and understanding whether there are differences in responses between PLWH and people not living with HIV may have implications for the development of diagnostic and surveillance algorithms, particularly in regions with high HIV burden.

To characterize the general B-cell response to a live SARS-CoV-2 isolate (B.1.1.117, referred to here as D614G to denote the only mutation of significance) representing a typical SARS-CoV-2 infecting virus in sub-Saharan Africa at the time of sampling, we report on the levels of anti–SARS-CoV-2 immunoglobulin (Ig) M, IgG, IgA, neutralizing antibodies, and antibody-secreting cells (ASCs) in a South African cohort of SARS-CoV-2–confirmed cases. We also disaggregate the study population based on HIV status to assess the impact of HIV on anti–SARS-CoV-2 antibody responses in PLWH on ART.

## METHODS

### Ethical Approval

The University of KwaZulu-Natal Institutional Review Board approved the study protocol. Written informed consent was obtained for all participants.

### Participant Enrollment

All study participants were aged >18 years, capable of giving informed consent, presented with a positive SARS-CoV-2 polymerase chain reaction (PCR)–based diagnosis, and were recruited from 2 hospitals (King Edward VIII and Clairwood) in Durban, KwaZulu-Natal, South Africa, between June 2020 and November 2020 (last enrollment date was 18 August 2020 after which no participants tested positive for SARS-CoV-2 by PCR, indicating that there were no reinfections). Participants consented to blood and nasopharyngeal/oropharyngeal swab collection at recruitment to the study and during weekly follow-up visits until day 28 and a further time point at 3 months post-enrollment. Each participant was subjected to a SARS-CoV-2 real-time quantitative (RT-qPCR) test that also served to quantify the SARS-CoV-2 viral load. Full genome SARS-CoV-2 sequencing was performed for only 16 participants (data not shown). All participants were ranked according to the World Health Organization ordinal scale for clinical improvement [20] as ambulatory, no limitation of activities; infected with limitation of activity; infected with limitation of activity and hospitalized; and infected, hospitalized, and supplemental oxygen provided (Table 1).

**Table 1.** Participant Enrollment Information

n, %	All (n = 72)	Participants without HIV (n = 42, 58.3%)	Participants with HIV (n = 30, 41.7%)	P Value (Stratified by HIV Status)
Female	55 (76.4%)	32 (76.2%)	23 (76.7%)	.9
Age, median (interquartile range), years	43.8 (33.3–51.9)	45.1 (32.5–54.9)	42.8 (34.9–50.5)	.5
Disease severity (ordinal scale^a^)				.883
Asymptomatic (1)	9 (12.5%)	6 (14.3%)	3 (10.0%)	
Mild (2/3)	45 (62.5%)	26 (61.9%)	19 (63.3%)	
Severe (4)	18 (25.0%)	10 (23.8%)	8 (26.7%)	
Comorbidity				
Hypertension	14 (19.4%)	9 (21.4%)	5 (16.7%)	.765
Diabetes	13 (18.1%)	10 (23.8%)	3 (10.0%)	.214
History of tuberculosis	8 (11.1%)	1 (2.4%)	7 (23.3%)	.008

Abbreviation: HIV, human immunodeficiency virus.

^a^Reference [20]

### RT-qPCR Detection of SARS-CoV-2

The QIAmp Viral RNA Mini kit (QIAGEN, Hilden, Germany) was used according to the manufacturer’s instructions to extract SARS-CoV-2 RNA. Three SARS-CoV-2 genes (ORF1ab, S, and N) were amplified using the TaqPath COVID-19 Combo kit and TaqPath COVID-19 CE-IVD RT-PCR kit (ThermoFisher Scientific, Waltham, MA) using a QuantStudio 7 Flex Real-Time PCR system and analyzed using the Design and Analysis software (ThermoFisher Scientific). Results were interpreted as positive if at least 2 of the 3 genes were amplified and regarded inconclusive if only 1 of the 3 genes was detected.

### Clinical Laboratory Testing

A separate blood sample per participant was sent to an accredited diagnostic laboratory (Molecular Diagnostic Services, Durban, KwaZulu-Natal, South Africa) for HIV testing by rapid test and quantification of HIV viral load. Blood CD4 and CD8 cell counts (cells per microliter) were performed by a commercial diagnostic laboratory (Ampath, Durban, South Africa; Supplementary Table 1).

### Receptor-Binding Domain Antibody Immune Assay

Collected plasma samples were tested for anti–SARS-CoV-2 IgM, IgG, and IgA antibodies as earlier described but with some modifications. Briefly, flat-bottom microplates (ThermoFisher Scientific) were coated with 500 ng/mL of the receptor-binding domain (RBD) of the spike protein from SARS-CoV-2 (GenBank: MN975262; provided by Dr Galit Alter from the Ragon Institute, Cambridge, MA) and incubated overnight at 4°C. Plates were blocked with a 200 µL/well tris-buffered saline containing 1% bovine serum albumin (TBSA) and incubated at room temperature for 1 hour. Samples were diluted in TBSA with .05% Tween-20 to 1:100, 1:1000, and 1:10 000. Subsequently, goat anti-human IgG (1:5000), IgM (1:5000), and IgA (1:10 000) horseradish peroxidase conjugated secondary antibodies (Jackson ImmunoResearch, West Grove, PA) were added to the respective plates (100 µL/well) and incubated at room temperature for 1 hour. Bound secondary antibodies were detected using 1-step Ultra TMB substrate (100 μL/well; ThermoFisher Scientific). Plates were incubated at room temperature for 3 (IgG) and 5 (IgM and IgA) minutes, respectively, in the dark before addition of 1 N sulfuric acid stop solution (100 μL/well). Plates were washed with high-salt TBS-containing .05% Tween-20 after each incubation. Standard curves were used to calculate the concentration (nanograms per milliliter) of anti-RBD expressed as IgG (anti–SARS-CoV-2 monoclonal, CR3022), IgM (anti–SARS-CoV-2 S1 RBD IgM, hIgM2001), or IgA (anti–SARS-CoV-2 S1 RBD IgA, hIgA2001; Genscript Piscataway, NJ) [21, 22]. We used prepandemic plasma samples as negative controls to define seroconversion cutoffs calculated as 3 times the standard deviation plus the mean of the negative samples for each isotype.

### Live Virus Microneutralization Assay

A live virus neutralization focus-forming assay using an isolate of SARS-CoV-2 (D614G) [23] was used with the following modifications: input virus was 100 focus-forming units per well and plasma samples were serially diluted 4-fold from 1:20 to 1:81 920 final concentrations. For each participant, 1 early (median days post-symptom onset, 30; interquartile range [IQR], 26–62) and 1 late (median, 102; IQR, 98–111) time point was selected. The dilution of sera required to inhibit viral infection by 50% relative to the no-antibody control wells (NT50) was calculated using a 4-parameter logistic curve fit (GraphPad Prism version 9.01).

### Quantification of Antibody-Secreting Cells

Blood was collected in EDTA tubes and diluted 1:3 with phosphate-buffered saline (PBS). Peripheral blood mononuclear cells (PBMCs) were isolated by density gradient centrifugation through Histopaque 1077 (Sigma-Aldrich) in SepMate separation tubes (STEMCELL Technologies, Vancouver, Canada). For immune phenotyping, 10^6^ fresh PBMCs were surface-stained in a 25-µL antibody mix containing a LIVE/DEAD fixable near-IR-dead cell-staining reagent (1:200 dilution, Invitrogen, Carlsbad, CA) with combinations of the listed antibodies from BD Biosciences (Franklin Lakes, NJ), BioLegend (San Diego, CA), or Beckman Coulter (Brea, CA). Cells were stained for 20 minutes in the dark at room temperature, followed by two 1-mL washes with cold PBS, then fixed in 2% paraformaldehyde and stored at 4°C until acquisition on a FACSAria Fusion III flow cytometer (BD Biosciences). CD19+ B cells were analyzed for the B-cell maturation markers CD27 and CD38 to identify ASCs (CD27 + CD38++) using FlowJo version 9.9.6 (Tree Star).

### Statistical Analyses

Statistical analyses were conducted in Stata (version 16) and GraphPad Prism (version 9.01). Lowess curves were generated to indicate kinetics of antibodies over time. Kaplan-Meier curves (multivariable analyses) were used to determine time to seroconversion, and the Pearson *χ*^2^ test was used to determine factors associated with the loss of antibodies, with 95% confidence intervals (CIs). Cox regression and the Breslow method for ties were used to determine any associations to antibody responses. The Spearman rank test was used to determine all correlations. *P* < .05 was considered significant.

## RESULTS

A total of 72 SARS-CoV-2–infected patients with 294 clinical specimens were analyzed in this longitudinal study. Full genome SARS-CoV-2 sequence data were available for 16 participants, none of whom were infected with a variant of concern (data not shown). Most participants were women (55 of 72, 76.4%), the median age was 43.4 years (IQR, 33.3–51.9), 30 of 72 (41.7%) were living with HIV, 25 of the 30 (83.0%) were on ART and fully HIV suppressed in the blood. CD4 cell counts were significantly lower (*P* = .0006) in PLWH, while CD8 cell counts were significantly higher (*P* = .0008) in PLWH. Hypertension was the leading comorbidity (19.4%) followed by diabetes (18.1%) and having a history of TB, which were significantly more common in PLWH (*P* = .008). Of the 72 participants, 6 (12.5%) had no clinical manifestation, 45 (62.5%) had mild clinical symptoms with limitation of activities, and 18 (25.0%) had severe clinical symptoms and required supplemental oxygen (Table 1). For all analyses, days from symptom onset was used; day of enrollment was used for asymptomatic cases.

**Figure 1.**
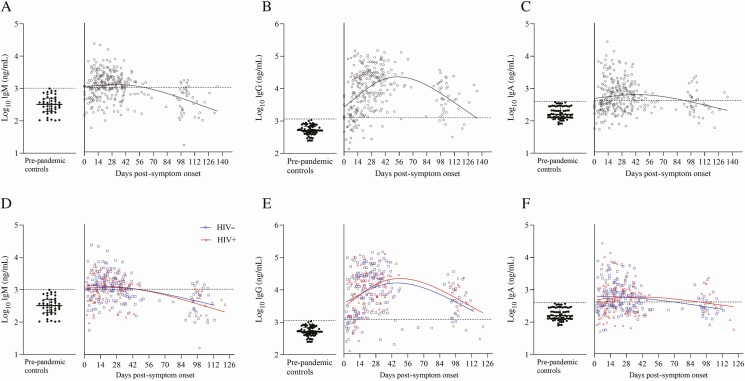
Effect of HIV status on temporal responses of different severe acute respiratory syndrome coronavirus 2–specific antibody isotypes. *A–C,* IgM, IgG, and IgA of all 72 participants (295 time points total) measured weekly up to 28 days and at 3 months post-symptom inset. *D–F,* Stratified according to people living with HIV (red) and people not living with HIV (blue). Locally weighted scatterplot smoothing curves were used to fit the data. Cutoffs for seroconversion are indicated on the *y*-axis with a dotted line at 3 log_10_ for IgM and IgG and at 2.6 log_10_ for IgA. Abbreviations: HIV, human immunodeficiency virus; Ig, immunoglobulin.

Anti–SARS-CoV-2 IgM, IgG, and IgA antibodies specific to the RBD were measured for all participants, sampled weekly up to 28 days with a further time point at 3 months (Figure 1A–1C). Overall, 67 of 72 (93.1%) participants had more than 1 measurement within 28 days, with the majority possessing SARS-CoV-2–specific IgM and IgG antibodies (53 of 72, 73.6% and 70 of 72 97.2%, respectively). SARS- CoV-2 IgA antibodies were less frequently observed but still present in most individuals (43 of 72, 59.7%; Figure 1A–1C). Samples were available for 43 of 72 (59.7%) participants at the 3-month time point post-enrollment. These samples showed a marked waning of the IgM (8 of 43,18.6%) and IgA (17 of 43, 39.5%) responses, but IgG responses were well maintained (37 of 43, 86.1%). Using the cutoffs defined earlier, we estimated the distribution of the time required to seroconversion for IgM, IgG, and IgA (Figure 1A–1C). The estimated median time to seroconversion was 17 days for IgM (IQR, 13–19), 13 days for IgG (IQR, 11–16), and 19 days for IgA (IQR, 16–31). Loss of IgA antibodies was significantly associated with age (*P* = .023) and having a history of TB (*P* = .0018), while no trend could be seen for IgM or IgG.

**Figure 2.**
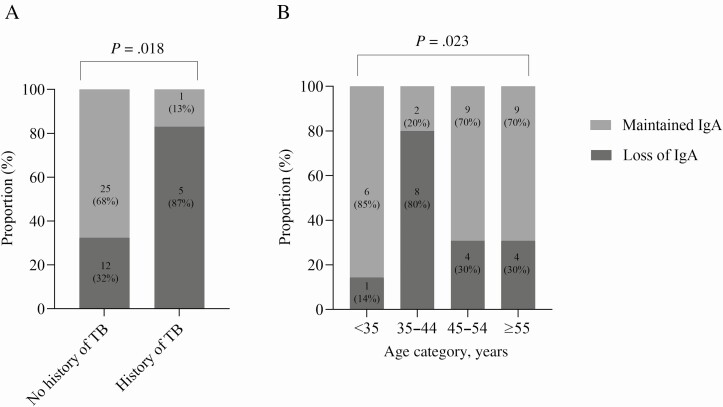
Loss of anti–severe acute respiratory syndrome coronavirus 2 IgA antibodies at 3 months post-symptom onset was significantly associated with having a history of TB (n = 6, *P* = .0018) *(A)* as well as age (n = 43, *P* = .023) *(B)*. Abbreviations: Ig, immunoglobulin; TB, tuberculosis.

Next, antibody responses were stratified by HIV status and overall; no differences in maximal anti–SARS-CoV-2 IgM, IgG, or IgA titers were observed (Figure 1D–1F). Early and acute antibody responses were similar between people not living with HIV and PLWH, with significantly higher responses during the convalescent weeks in PLWH (weeks 8, 7, and 7 for IgM (*P* = .0455), IgG (*P* = .0133), and IgA (*P* = .0133), respectively; Supplementary Figure 1). There was also no difference in time to seroconversion between PLWH and participants not living with HIV for any isotype. HIV-associated parameters (HIV plasma viral load, CD4 and CD8 cell counts, and CD4:CD8 ratio) were also not associated with significant antibody losses in PLWH across all 3 isotypes. A history of TB (only 6 participants with a history of TB developed IgA; *P* = .0018) and age (34–45 years; *P* = .023) were associated with loss of IgA antibodies at 3 months post-symptom onset (Figure 2A, 2B), while no trend was noted for IgM or IgG.

**Figure 3.**
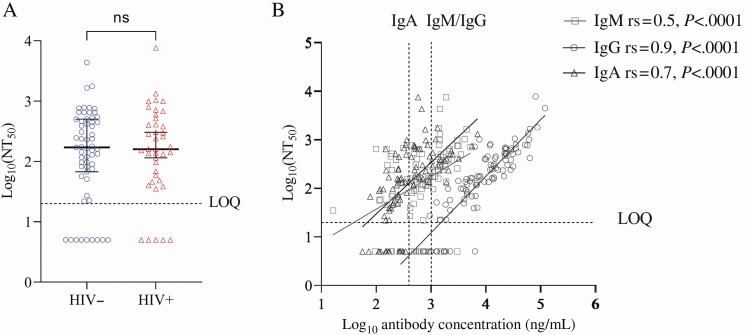
Neutralization by convalescent plasma of D614G severe acute respiratory syndrome coronavirus 2 (SARS-CoV-2) using a live virus neutralization assay. *A,* Neutralization measured as NT50 in participants not living with HIV (blue) and people living with HIV (PLWH; red). The horizontal line indicates the most concentrated plasma dilution tested in the assay. Shown is median and interquartile range for n = 59 participants not living with HIV and n = 40 PLWH. *B,* Correlation between SARS-CoV-2 anti-IgG and IgA binding and neutralization capacity. Abbreviations: HIV, human immunodeficiency virus; Ig, immunoglobulin; LOQ, limit of quantification; ns, not significant; NT, neutralization titer; rs, Spearman’s correlation coefficient.

**Figure 4.**
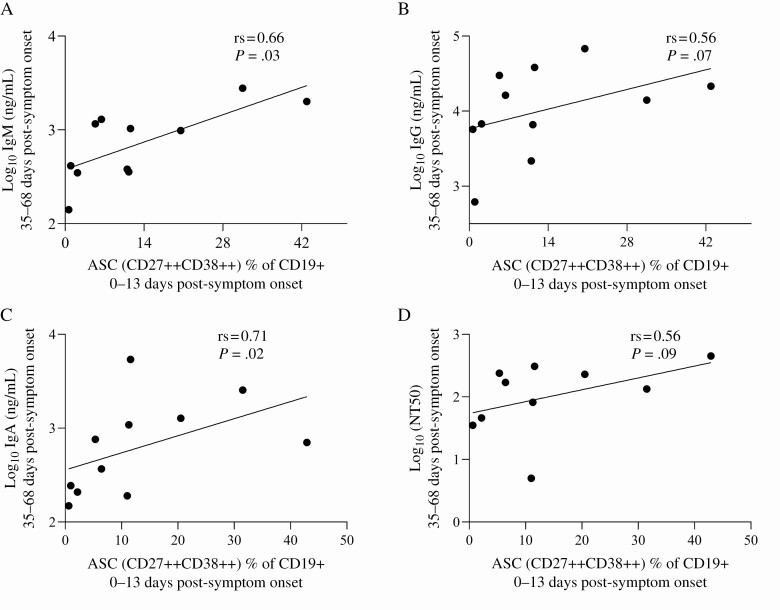
Correlation between frequency of antibody-secreting cells and antibody isotypes. Concentrations of anti–severe acute respiratory syndrome coronavirus 2 IgM *(A)*, IgG *(B)*, IgA *(C)*, and neutralizing antibody *(D)* production 35–68 days post-symptom onset as a function of the frequency of ASCs 0–13 days post-symptom onset. Abbreviations: ASC, antibody-secreting cell; Ig, immunoglobulin; rs, Spearman’s correlation coefficient.

Using a live virus focus-forming assay to measure anti–SARS-CoV-2 neutralization (NT50), we observed no difference in neutralizing potency between PLWH and participants not living with HIV (Figure 3A). Anti–SARS-CoV-2 neutralization positively correlated most strongly with IgG titers from the same time points (r_s_ = .9, *P* < .001), followed by IgA (r_s_ = .7, *P* < .001) and IgM (r_s_ = .5, *P* < .001; Figure 3B). No significant differences in the neutralization ability were detected between PLWH and participants not living with HIV. To examine the cellular correlates of neutralizing antibodies, we measured the frequency of CD27 + CD38++ ASC at baseline in a subset of individuals using flow cytometry (Figure 4A–4D). ASC, measured at 0–13 days post-symptom onset, significantly correlated with IgM (r_s_ = .66, *P* = .03) and IgA (r_s_ = .71, *P* = .02) titers with a similar correlation observed for IgG (r_s_ = .56, *P* = .07) and neutralizing (r_s_ = .56, *P* = .09) antibodies measured at 35–68 days post-symptom onset.

**Figure 5.**
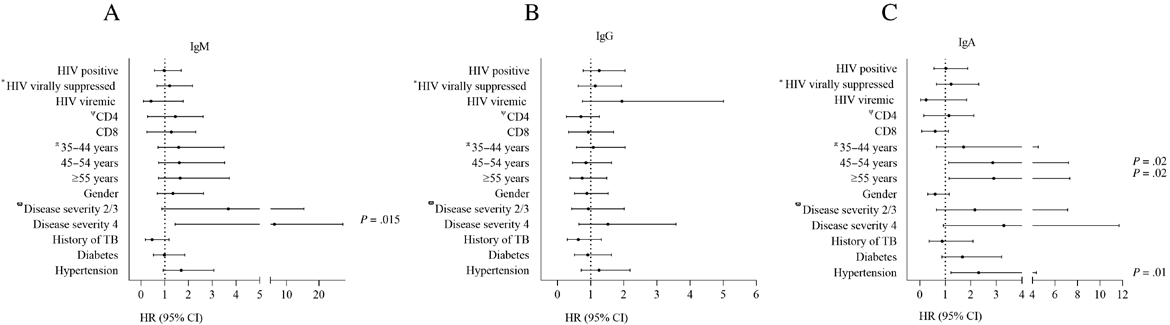
HRs for covariates that impact anti–severe acute respiratory syndrome coronavirus 2 antibody production. HRs and 95% CIs for IgM *(A)*, IgG *(B)*, and IgA *(C)* with significant associations indicated at *P* < .05. *HIV virally suppressed and HIV viremic participants are compared with participants not living with HIV. ^Ψ^Association of antibody responses with CD4 and CD8 cell counts in participants living with HIV. ^Π^ Association of age groups with antibody responses (participants aged <35 years are the reference group). ^ϖ^ Association of antibody responses with disease severity (asymptomatic participants; disease severity score 1 is the reference group). Abbreviations: CI, confidence interval; HIV, human immunodeficiency virus; HR, hazard ratio; Ig, immunoglobulin; TB, tuberculosis.

Finally, we tested the relationship between SARS-CoV-2–specific antibodies and clinical parameters using regression analysis. Anti–SARS-CoV-2 IgM antibody responses were significantly associated with disease severity and the need for supplemental oxygen (hazard ratio [HR] IgM, 6.3; 95% CI, 1.4–27.4; *P* = .015; Figure 5A). IgA antibody production was significantly associated with older age (45–54 years: HR, 2.9; 95% CI, 1.1–7.2; *P* = .02 and >55 years: HR, 2.9; 95% CI, 1.2–7.3; *P* = .02) and hypertension (HR, 2.3; (95% CI, 1.2–4.4; *P* = .01; Figure 5C). IgG antibody production was not associated with any clinical covariates. Strikingly, and in line with the data presented, HIV coinfection (negative vs positive), HIV viral load (not living with HIV vs HIV virally suppressed and HIV viremic), and CD4 and CD8 cell counts were not associated with differences in any of the antibody isotypes measured (Figure 5).

## DISCUSSION

Here, we report on longitudinal anti–SARS-CoV-2 IgM, IgG, and IgA antibody titers and neutralization activity in a sub-Saharan African population with a high burden of HIV. There were no differences in IgM, IgG, or IgA kinetics, durability, or neutralization potency between people not living with HIV and PLWH, 83% of whom were fully HIV suppressed. These data agree with recently published data that showed similar seroconversion kinetics in PLWH with well-controlled disease and people not living with HIV [24–27], highlighting the importance of a secure supply of ART for PLWH during the COVID-19 pandemic. Overall, a lack of individuals with unsuppressed HIV were available to determine its effect of uncontrolled HIV in SARS- CoV-2 serology. Nonetheless, these data demonstrate that HIV coinfection per se does not appear to limit the initial antibody response in the first SARS-CoV-2 infection wave in South Africa. Numerous studies have demonstrated the effect of ART on vaccine-modulated immunity against polio and measles. As such, a similar benefit of ART could lead to the observed similarities between individuals living with and without HIV in this study.

Overall, age was significantly associated with both production and loss of IgA. This trend, however, was not observed when stratified by HIV status, probably a limitation of the sample size. Interestingly, loss of IgA was also associated with a previous diagnosis of TB. Current and previous diagnoses of TB have also been associated with COVID-19 death in a population cohort study in South Africa [9]. In other studies, active TB disease has been associated with a lower frequency of B cells and a higher frequency of atypical double-negative B cells; whereas when the frequency of total B cells normalized after treatment, individuals with a history of TB treatment still had higher double-negative B cells [28]. Therefore, active TB might affect the humoral response to other pathogens including SARS-CoV-2. Since *Mycobacterium tuberculosis* and SARS-CoV-2 are both respiratory pathogens, existing TB-related damage to the mucous and respiratory membranes could also be further exacerbated by SARS-CoV-2 infection and affect mucosal immunity. These potential interactions between respiratory pathogens need further investigation. Antibody-secreting cells at 0–13 days post-symptom onset positively correlated with IgG and IgA production 35–68 days post-symptom onset. Due to the small sample size, we could not stratify into individuals living with and without HIV. A recent study, however, reported no difference in ASCs between these 2 groups [29].

Multiple studies have shown that most SARS-CoV-2–infected individuals produce S- and RBD-specific antibodies during the primary response. RBD-specific monoclonal antibodies can neutralize the virus in vitro and in vivo [30]. A recent study demonstrated higher sensitivity of the S protein vs the nucleocapsid protein for both the acute and post-infection phases with the anti-N IgG antibodies waning after acute infection [18]. Therefore, RBD-specific antibodies would likely contribute to protection against reinfection. These results are similar to those from previous reports. Whether the neutralization response is also similar in subsequent infection waves with variants that show multiple functional differences to the original circulating strains has yet to be determined.

There were limitations to the study. First, the sample size of HIV viremic participants was small. Only 5 people were HIV viremic; therefore, we can only conclude on the effect of HIV on antibody kinetics in ART virally suppressed individuals, as well as on the impact that HIV-associated parameters such as CD4 cell counts have on antibody responses. Future studies should focus on HIV suppressed vs HIV viremic patients. Second, sample base-line blood at enrollment was taken between 0 and 37 days post-symptom onset. Consequently, we may have missed the peak IgM response in some individuals, and time to seroconversion may be shorter than reported here. Third, only 16 of 72 SARS-CoV-2 full genomes were available; therefore, we were unable to confirm the infecting viral variants. However, it should be noted that all participants were enrolled into the study by mid-August 2020, prior to the circulation of the Beta variant of concern (beta VOC). Last, the associations between loss of IgA antibodies, TB, and age should be interpreted with caution due to the small sample size.

Despite these caveats, these data show similar antibody kinetics, durability, and neutralization potency in PLWH on stable ART and people not living with HIV for SARS-CoV-2 D614G during the first wave of infections in an African setting. This provides hope that COVID-19 vaccines will be effective in PLWH on stable ART.

## Supplementary Data

Supplementary materials are available at *Clinical Infectious Diseases* online. Consisting of data provided by the authors to benefit the reader, the posted materials are not copyedited and are the sole responsibility of the authors, so questions or comments should be addressed to the corresponding author.

ciab758_suppl_Supplementary_Data

## Notes

***Acknowledgments.*** The authors thank the study participants, their clinicians, and members of the collaborating teams in the COMMIT-KZN consortium.

***Disclaimer.*** The views expressed here are those of the author(s) and not necessarily those of the African Academy of Sciences (AAS), New Partnership for Africa’s Development Planning and Coordinating (NEPAD) Agency, Wellcome Trust, or the UK government.

***Financial support.*** This work was supported by a grant from the Wellcome Trust to the Africa Health Research Institute (Wellcome Trust Strategic Core Award; grant 201433/Z/16/Z). Additional funding was received from the Bill and Melinda Gates Foundation (grant INV-018944), the South African Department of Science and Innovation through the National Research Foundation (South African Research Chairs Initiative; grant 64809), and the Victor Daitz Foundation. Additional funding was provided by the Sub-Saharan African Network for TB/HIV Research Excellence, a DELTAS Africa Initiative (grantDEL-15–006). The Developing Excellence in Leadership, Training and Science (DELTAS) Africa Initiative is an independent funding scheme of the AAS Alliance for Accelerating Excellence in Science in Africa and supported by the NEPAD Agency with funding from the Wellcome Trust (grant107752/Z/15/Z) and the UK government.

***Potential conflicts of interest.*** All authors: No reported conflicts of interest. All authors have submitted the ICMJE Form for Disclosure of Potential Conflicts of Interest. Conflicts that the editors consider relevant to the content of the manuscript have been disclosed.

**COMMIT-KZN Team**

Moherndran Archary^1^, Kaylesh J Dullabh^2^, Philip Goulder^3,4^, Guy Harling^3,5^, Rohen Harrichandparsad^6^, Kobus Herbst^3,7^, Prakash Jeena^1^, Thandeka Khoza^3^, Nigel Klein^3,8^, Henrik Kløverpris^3,9,10^, Rajhmun Madansein^2^, Mohlopheni Marakalala^3,9^, Matilda Mazibuko^3^, Mosa Moshabela^11^, Ntombifuthi Mthabela^3^, Kogie Naidoo^12^, Zaza Ndhlovu^3,13^, Kennedy Nyamande^14^, Nesri Padayatchi^12^, Vinod Patel^15^, Theresa Smit^3^, Adrie Steyn^3,16^, Emily Wong^3,16^

^1^Department of Paediatrics and Child Health, University of KwaZulu-Natal, Durban, South Africa; ^2^Department of Cardiothoracic Surgery, University of KwaZulu-Natal, Durban, South Africa; ^3^Africa Health Research Institute, Durban, South Africa; ^4^Department of Paediatrics, Oxford, United Kingdom; ^5^Institute for Global Health, University College London, United Kingdom; ^6^Department of Neurosurgery, University of KwaZulu-Natal, Durban, South Africa; ^7^South African Population Research Infrastructure Network, Durban, South Africa; ^8^Institute of Child Health, University College London, United Kingdom; ^9^Division of Infection and Immunity, University College London, London, United Kingdom; ^10^Department of Immunology and Microbiology, University of Copenhagen, Copenhagen, Denmark; ^11^College of Health Sciences, University of KwaZulu-Natal, Durban, South Africa; ^12^Centre for the AIDS Programme of Research in South Africa, Durban, South Africa; ^13^Ragon Institute of Massachusetts General Hospital (MGH), Massachusetts Institute of Technology (MIT) and Harvard, Boston, Massachusetts, USA; ^14^Department of Pulmonology and Critical Care, University of KwaZulu-Natal, Durban, South Africa; ^15^Department of Neurology, University of KwaZulu-Natal, Durban, South Africa; ^16^Division of Infectious Diseases, University of Alabama–Birmingham, Birmingham, Alabama, USA

## References

[1] GBD. Global Burden of Diseases. 2021. Available at: https://vizhub.healthdata.org/gbd-compare/. Accessed 19 May 2021.

[2] Pasin C , Balelli I, Van Effelterre T, et al Dynamics of the humoral immune response to a prime-boost Ebola vaccine: quantification and sources of variation. J Virol 2019; 93:e00579–19. 31243126 10.1128/JVI.00579-19 PMC6714808

[3] Muyanja E , Ssemaganda A, Ngauv P, et al Immune activation alters cellular and humoral responses to yellow fever 17D vaccine. J Clin Invest 2014; 124:3147–58. 24911151 10.1172/JCI75429 PMC4071376

[4] World Health Organization. HIV/AIDS: disease burden. 2021. Available at: https://www.afro.who.int/health-topics/hivaids. Accessed 19 May 2021.

[5] UNAIDS. Country factsheets: South Africa—2019. 2021. Available at: https://www.unaids.org/en/regionscountries/countries/southafrica. Accessed 19 May 2021.

[6] Nabatanzi R , Bayigga L, Cose S, et al Monocyte dysfunction, activation, and inflammation after long-term antiretroviral therapy in an African cohort. J Infect Dis 2019; 220:1414–9. 31323092 10.1093/infdis/jiz320 PMC6761975

[7] Wada NI , Jacobson LP, Margolick JB, et al The effect of HAART-induced HIV suppression on circulating markers of inflammation and immune activation. AIDS 2015; 29:463–71. 25630041 10.1097/QAD.0000000000000545 PMC4311407

[8] Bhaskaran K , Rentsch CT, MacKenna B, et al HIV infection and COVID-19 death: a population-based cohort analysis of UK primary care data and linked national death registrations within the OpenSAFELY platform. Lancet HIV 2021; 8:e24–32. 33316211 10.1016/S2352-3018(20)30305-2 PMC7773630

[9] Boulle A , Davies MA, Hussey H, et al Risk factors for COVID-19 death in a population cohort study from the Western Cape Province, South Africa. Clin Infect Dis 2020; ciaa1198. 10.1093/cid/ciaa1198 PMC7499501 32860699

[10] Tesoriero JM , Swain CE, Pierce JL, et al COVID-19 outcomes among persons living with or without diagnosed HIV infection in New York State. JAMA Netw Open 2021; 4:e2037069. 33533933 10.1001/jamanetworkopen.2020.37069 PMC7859843

[11] Khoury DS , Cromer D, Reynaldi A, et al Neutralizing antibody levels are highly predictive of immune protection from symptomatic SARS-CoV-2 infection. Nat Med 2021; 27:1205–11. 34002089 10.1038/s41591-021-01377-8

[12] Lane HC , Masur H, Edgar LC, Whalen G, Rook AH, Fauci AS. Abnormalities of B-cell activation and immunoregulation in patients with the acquired immunodeficiency syndrome. N Engl J Med 1983; 309:453–8. 6224088 10.1056/NEJM198308253090803

[13] Pensieroso S , Cagigi A, Palma P, et al Timing of HAART defines the integrity of memory B cells and the longevity of humoral responses in HIV-1 vertically-infected children. Proc Natl Acad Sci U S A 2009; 106:7939–44. 19416836 10.1073/pnas.0901702106 PMC2683072

[14] Helfand RF , Witte D, Fowlkes A, et al Evaluation of the immune response to a 2-dose measles vaccination schedule administered at 6 and 9 months of age to HIV-infected and HIV-uninfected children in Malawi. J Infect Dis 2008; 198:1457–65. 18828743 10.1086/592756

[15] Moir S , Buckner CM, Ho J, et al B cells in early and chronic HIV infection: evidence for preservation of immune function associated with early initiation of antiretroviral therapy. Blood 2010; 116:5571–9. 20837780 10.1182/blood-2010-05-285528 PMC3031405

[16] Muema DM , Macharia GN, Hassan AS, et al Control of viremia enables acquisition of resting memory B cells with age and normalization of activated B cell phenotypes in HIV-infected children. J Immunol 2015; 195:1082–91. 26116511 10.4049/jimmunol.1500491 PMC4505960

[17] Gelinck LB , Jol-van der Zijde CM, Jansen-Hoogendijk AM, et al Restoration of the antibody response upon rabies vaccination in HIV-infected patients treated with HAART. AIDS 2009; 23:2451–8. 19741483 10.1097/QAD.0b013e328331a43b

[18] Fenwick C , Croxatto A, Coste AT, et al Changes in SARS-CoV-2 spike versus nucleoprotein antibody responses impact the estimates of infections in population-based seroprevalence studies. J Virol 2021; 95:e01828–20. 10.1128/JVI.01828-20 PMC7925109 33144321

[19] Uyoga S , Adetifa IMO, Karanja HK, et al Seroprevalence of anti-SARS-CoV-2 IgG antibodies in Kenyan blood donors. Science 2021; 371:79–82. 33177105 10.1126/science.abe1916 PMC7877494

[20] World Health Organization. *COVID-19 Therapeutic Trial Synopsis.* Available at: https://www.who.int/blueprint/priority-diseases/key-action/COVID-19_Treatment_Trial_Design_Master_Protocol_synopsis_Final_18022020.pdf. Accessed 2 June 2021.

[21] Iyer AS , Jones FK, Nodoushani A, et al Persistence and decay of human antibody responses to the receptor binding domain of SARS-CoV-2 spike protein in COVID-19 patients. Sci Immunol 2020; 5:eabe0367. 33033172 10.1126/sciimmunol.abe0367 PMC7857394

[22] Wang C , Li W, Drabek D, et al Publisher correction: a human monoclonal antibody blocking SARS-CoV-2 infection. Nat Commun 2020; 11:2511. 32409714 10.1038/s41467-020-16452-w PMC7224291

[23] Cele S , Gazy I, Jackson L, et al Escape of SARS-CoV-2 501Y.V2 from neutralization by convalescent plasma. Nature 2021; 593:142–6. 10.1038/s41586-021-03471-w PMC9867906 33780970

[24] Yamamoto S , Saito M, Nagai E, et al Antibody response to SARS-CoV-2 in people living with HIV. J Microbiol Immunol Infect 2021; 54:144–6. 33046418 10.1016/j.jmii.2020.09.005 PMC7531336

[25] Pallikkuth S , Sharkey M, Beauchamps L, et al SARS-COV-2–specific AB response in HIV+ individuals on ART. Virtual Conference on Retroviruses and Opportunistic Infections. San Francisco, CA: International Antiviral Society, 2021.

[26] Alrubayyi A , Gea-Mallorqui E, Touizer E, et al Characterization of SARS-CoV-2–specific responses in people living with HIV. Virtual Conference on Retroviruses and Opportunistic Infections. San Francisco, CA : International Antiviral Society, 2021.

[27] Cooper TJ , Woodward BL, Alom S, Harky A. Coronavirus disease 2019 (COVID-19) outcomes in HIV/AIDS patients: a systematic review. HIV Med 2020; 21:567–77. 32671970 10.1111/hiv.12911 PMC7405326

[28] Joosten SA , van Meijgaarden KE, Del Nonno F, et al Patients with tuberculosis have a dysfunctional circulating B-cell compartment, which normalizes following successful treatment. PLoS Pathog 2016; 12:e1005687. 27304615 10.1371/journal.ppat.1005687 PMC4909319

[29] Karim F , Gazy I, Cele S, et al HIV infection alters SARS-CoV-2 responsive immune parameters but not clinical outcomes in COVID-19 disease. medRxiv 2020; doi:2020.11.23.20236828v1.

[30] Shi R , Shan C, Duan X, et al A human neutralizing antibody targets the receptor-binding site of SARS-CoV-2. Nature 2020; 584:120–4. 32454512 10.1038/s41586-020-2381-y

