# Supplementary material for: Similar Antibody Responses Against Severe Acute Respiratory Syndrome Coronavirus 2 in Individuals Living Without and With Human Immunodeficiency Virus on Antiretroviral Therapy During the First South African Infection Wave
Source: Clin Infect Dis. 2021 Sep 2;75(1):e249–56. doi: 10.1093/cid/ciab758 (PMC8522359; doi:10.1093/cid/ciab758)
Supplement: ciab758_suppl_Supplementary_Data [file ciab758_suppl_supplementary_data.docx]

**Supplementary Table S1:** HIV positive participants enrolled in this study with baseline SARS-CoV-2 viral load, HIV viral load, CD4, CD8 and CD4:CD8 ratio as well as reported TB history.

| **Patient ID** | **SARS-CoV-2 Viral load** | **HIV Viral Load** | **CD4 (cells/uL)** | **CD8 (cells/uL)** | **CD4:CD8 Ratio** | **History of TB** |
| --- | --- | --- | --- | --- | --- | --- |
| 039-02-0009 | 30.5 | 40 | 706 | 833 | 0.8 | No |
| 039-02-0011 | 28.35 | 40 | 176 | 473 | 0.4 | No |
| 039-02-0015 | 18.1 | 40 | 201 | 644 | 0.3 | Yes |
| 039-02-0016 | >34.1 | 40 | 311 | 1080 | 0.3 | No |
| 039-02-0018 | Not Detected | 40 | 497 | 785 | 0.6 | No |
| 039-02-0019 | Not Detected | 22904 | 8 | 455 | 0.0 | Yes |
| 039-02-0021 | Not Detected | 40 | 663 | 546 | 1.2 | No |
| 039-02-0025 | 37.2 | 2862 | 126 | 287 | 0.4 | No |
| 039-13-0003 | Not Detected | 40 | 1598 | 1128 | 1.4 | No |
| 039-13-0004 | 20.3 | 40 | 544 | 467 | 1.2 | No |
| 039-13-0009 | >36.7 | 40 | 1134 | 941 | 1.2 | No |
| 039-13-0010 | 20.4 | 40 | 703 | 637 | 1.1 | No |
| 039-13-0013 | 18.2 | 40 | 306 | 386 | 0.8 | Yes |
| 039-13-0019 | Not Detected | 177218 | 175 | 700 | 0.3 | No |
| 039-13-0027 | Not Detected | 40 | 1118 | 520 | 2.2 | No |
| 039-13-0028 | Inconclusive | 8634 | 473 | 977 | 0.5 | No |
| 039-13-0031 | Inconclusive | 40 | 942 | 1135 | 0.8 | No |
| 039-13-0032 | Not Detected | 40 | 747 | 830 | 0.9 | Yes |
| 039-13-0035 | Not Detected | 40 | 598 | 601 | 1.0 | Yes |
| 039-13-0036 | Not Detected | 40 | 570 | 534 | 1.1 | No |
| 039-13-0037 | 28.9 | 40 | 585 | 1013 | 0.6 | No |
| 039-13-0039 | Inconclusive | 40 | 467 | 1117 | 0.4 | No |
| 039-13-0045 | Not Detected | 40 | 382 | 658 | 0.6 | No |
| 039-13-0046 | 30.6 | 40 | 913 | 768 | 1.2 | No |
| 039-13-0052 | Not Detected | 164 | 274 | 1462 | 0.2 | Yes |
| 039-13-0054 | >36.6 | 40 | 612 | 467 | 1.3 | No |
| 039-13-0060 | 30.5 | 40 | 715 | 1178 | 0.6 | No |
| 039-13-0063 | 34.2 | 40 | 306 | 216 | 1.4 | No |
| 039-13-0064 | Inconclusive | 40 | 457 | 735 | 0.6 | Yes |
| 039-13-0069 | Inconclusive | 40 | 702 | 817 | 0.9 | No |


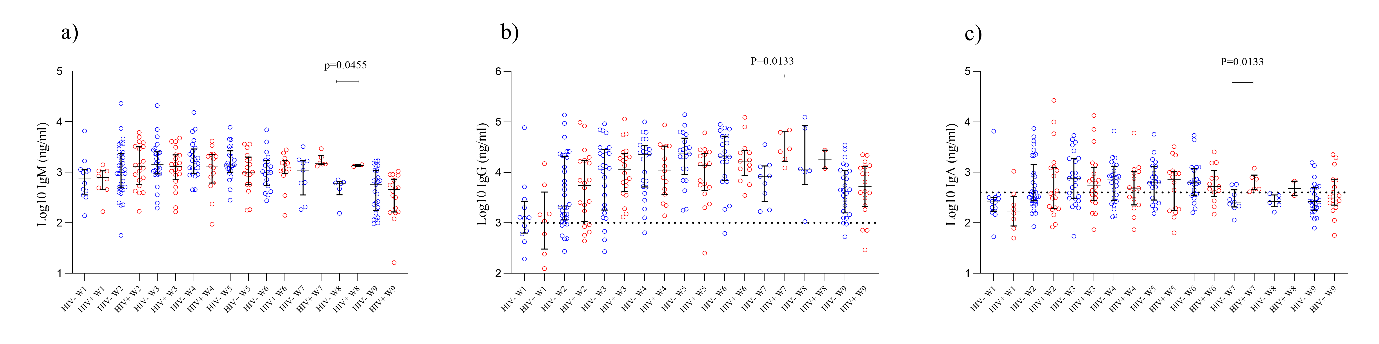


**Supplementary Figure S1:**

Effect of HIV status on temporal responses of different anti-SARS-CoV-2 specific antibody isotypes. (a-c) IgM, IgG, and IgA per week stratified according to PLWH (indicated in red) and HIV negative (indicated in blue). Significant higher antibody responses were observed during the convalescent weeks in PLWH (weeks 8, 7 and 7 for IgM (p=0.0455), IgG (p=0.0133), and IgA (p=0.0133) respectively). Cut-offs for seroconversion are indicated on the y-axis with a dotted line at 3 log_10_ for IgM and IgG and 2.6 log_10_ for IgA.
